# How the First Year of COVID-19 Affected Elective Pediatric Urology Patients: A Longitudinal Study Based on Waiting Lists and Surveys From 10 European Centers

**DOI:** 10.3389/fpubh.2022.874758

**Published:** 2022-04-28

**Authors:** Nikolai Juul, Aurélie Cazals, Aybike Hofmann, Virginia Amesty, Gilvydas Verkauskas, Barbara Dobrowolska-Glazar, Gundela Holmdahl, Maria Escolino, Jacques Birraux, Tamas Kovacs, Nicolas Kalfa, Magdalena Fossum

**Affiliations:** ^1^Division of Pediatric Surgery, Department of Surgery and Transplantation, Rigshospitalet Copenhagen University Hospital, Copenhagen, Denmark; ^2^Service de Chirurgie Viscérale et Urologique Pédiatrique, Hôpital Lapeyronie, CHU de Montpellier, Montpellier, France; ^3^Department of Pediatric Urology, KUNO Clinic St. Hedwig, University Medical Center, Regensburg, Germany; ^4^Department of Pediatric Urology, Hospital Universitario La Paz, Madrid, Spain; ^5^Children's Surgery, Orthopedics and Traumatology Center, Faculty of Medicine, Vilnius University, Vilnius, Lithuania; ^6^Department of Pediatric Urology, Jagiellonian University Medical College, Krakow, Poland; ^7^Department of Pediatric Surgery, Karolinska University Hospital, Stockholm, Sweden; ^8^Department of Women's and Children's Health, Karolinska Institutet, Stockholm, Sweden; ^9^Pediatric Surgery and Urology Unit, Federico II University Hospital, Naples, Italy; ^10^Service de Chirurgie de l'Enfant et de l'Adolescent, Centre Universitaire romand de Chirurgie Pédiatrique, Hôpitaux Universitaire de Genève, Genève, Switzerland; ^11^Division of Pediatric Surgery, Department of Pediatrics, Albert Szent-Gyorgyi Clinical Center, University of Szeged, Szeged, Hungary; ^12^Université de Montpellier, Institut Debrest de Santé Publique IDESP, UMR INSERM, Montpellier, France

**Keywords:** pediatrics, urology, waiting lists, COVID-19, multicenter study

## Abstract

**Introduction:**

COVID-19 impacted healthcare systems worldwide, and elective surgical activity was brought to a minimum. Although children were not primarily affected by the disease, pediatric urology was halted by clinical closedown and staff allocation. We aimed to document how these prioritizations affected waiting lists, and to investigate how European centers dealt with the challenge of these logistical and financial prioritizations.

**Materials and Methods:**

This was a 1-year prospective study, starting March 2020. Participants were surveyed at 3-month intervals about waiting lists for several common procedures as well as OR capacity and funding. Further, centers retrospectively reported on surgical and outpatient activity rates during 2019–2021. Waiting list tendencies were evaluated in relation to study baseline.

**Results:**

A marked decrease in surgical and outpatient activity was seen in the spring of 2020. Some included pediatric urology centers were able to increase their budget (15%) and staff working hours (20%) during part of the study period. Still, at the end of the study, the centers had increased the total number of patients on waiting lists with 11%, whereas the average days on waiting lists had accumulated with 73%, yielding a total of 6,102 accumulated waiting days in the study population. Centers with decreased resources had markedly negative effects on waiting lists.

**Conclusions:**

Correlations between COVID-19 derived burdening of healthcare systems and the availability of pediatric urology greatly depends on the prioritizations made at individual centers. Ongoing monitoring of these correlations is warranted to safely avoid unnecessary negative impact on the pediatric population.

## Introduction

Coronavirus disease 2019 (COVID-19) deeply impacted the activity of pediatric urology cases in Europe and has had consequences during an extended period of time. Early on in the pandemic, statements and guidelines from European and North American pediatric urological societies recommended only to perform surgery in cases of organ- or life-threatening disease during lockdown, and further suggested to reduce all outpatient clinic consultations during the first wave of the COVID-19 pandemic (1–3). New protocols have been established to adopt minimally invasive surgery to treat pediatric surgical and urological pathologies during this pandemic, aiming at preserving both patient's and surgeon's safety (4, 5). While these guidelines were structured to prioritize cases in relation to urgency, clinics were also facing the challenge of complying with and taking in to account the varying degrees of less urgent, however already heavily surceased, elective procedures (6).

This initiative was undertaken in order to launch a collaborative European multicenter study on the COVID-19 pandemic and how this has affected pediatric urology cases and patients related to the ERN eUROGEN work stream 1 disease areas. The primary aim of the study was to evaluate how closure of elective interventions affected the pediatric urology population by increased time and patients on the waiting list for surgical interventions. The secondary aim was to evaluate how different centers dealt with the problem of an increased caseload before and after re-opening.

By prospectively gathering data from European centers, we intended to provide an overview of the current impacts which could be used as a tool for information and political decision-making at a local, regional, or European level. Since the COVID-19 pandemic is far from over, and since new pandemics may very well arise, this paper is meant to help in the future planning for dealing with pandemics or other major crises affecting societies and health care.

## Materials and Methods

This was a European multicenter study comprising 10 tertiary pediatric urological centers broadly representing most parts of western Europe (Table 1). Centers were recruited by asking representatives from all the health care providers that were part of the European reference network eUROGEN work stream 1 (rare congenital urogenital and rectal anomalies) or by direct contact with European centers fulfilling the European reference network inclusion criteria (7).

**Table 1:** Participating centers with corresponding country codes and reference populations in million people.

**Center**	**City (country)**	**Ref. population (mio.)**
Clinic St. Hedwig University Medical Center	Regensburg (DE)	1.2
Federico II University Hospital	Naples (IT)	1.5
Vilnius University Hospital Santaros Klinikos	Vilnius (LI)	1.5
Albert Szent-Gyorgyi Clinical Center	Szeged (HU)	1.5
Hospital Universitario La Paz	Madrid (ES)	2
University Center of Pediatric Surgery of Western Switzerland	Geneva (CH)	2.4
Rigshospitalet University Hospital	Copenhagen (DK)	2.5
Hôpital Lapeyronie CHU de Montpellier	Montpellier (FR)	2.7
Karolinska University Hospital	Stockholm (SE)	3
Jagiellonian University Medical College	Krakow (PL)	3.3

To be included in the study, the participating center would have to be a tertiary referral center performing advanced pediatric urology cases, although not by definition exclusively. Centers should have access to pediatric intensive care units, a minimum of 1 million population base for referrals of specialized pediatric urology and/or pediatric surgery cases, and in an area where COVID-19 closedown required cancellations of all elective surgery (only running emergency and imperative cases). Chief of department in all centers approved retrieval of information regarding waiting lists to surgery and data collection, and surveys were compiled and answered by the co-author representing each center. All reported data from waiting lists was anonymized on a patient level.

The study design was a longitudinal, prospective study based on pediatric urology cases waiting lists in different European centers. Centers participated over a 13-months study period (March 2020 to April 2021). At five timepoints (March, June, October, January, and April, respectively), centers were asked to count the waiting lists within a range of predetermined elective, non-emergent and non-oncologic urological procedures (Table 2). To differentiate our results further, centers were asked to report on both the number of patients waiting and the accumulated days waiting for each procedure. Averaged cross-country European COVID-19 numbers were included as an intuitive reference to the broadscale implications of the pandemic. Data on nationwide new weekly COVID-19 admissions and completed vaccinations were gathered from the publications of the European Center for Disease Prevention and Control (8).

**Table 2:** Total number of patients and mean days per person on waiting lists, for each procedure of interest and across all participating centers, presented in absolute numbers.

	**March 2020 (T0)**		**June 2020 (T1)**		**October 2020 (T2)**		**January 2021 (T3)**		**April 2021 (T4)**	
**Intervention**	**Patients**	**Days**	**Patients**	**Days**	**Patients**	**Days**	**Patients**	**Days**	**Patients**	**Days**
Hypospadias repair	298	142	298	185	311	167	317	176	272	193
Orchidopexy	257	75	228	109	275	104	314	95	274	110
Foreskinplasty (phimosis)	183	80	178	124	199	116	256	96	247	137
Cystoscopy (diagnostic or therapeutic)	85	79	92	114	76	96	91	118	89	127
Pyeloplasty (open or laparoscopic)	18	55	18	60	18	36	19	47	15	34
Heminephrectomy or nephrectomy (open)	10	90	13	96	8	104	15	65	13	51
Surgery of the kidney (laparoscopic or robotic)	4	36	2	119	4	38	5	66	6	7
Bladder surgery for ureteric pathologies (open)	49	76	30	124	26	118	30	102	32	79
Intervention for urolithiasis	3	20	4	66	7	122	9	48	8	78
Bladder exstrophy closure	1	30	3	77	3	148	5	50	1	49
Epispadias surgery	5	142	8	155	6	197	9	158	9	144
Surgery of the bladder (laparoscopic or robotic)	3	60	2	121	1	16	3	47	4	150
Bladder neck surgery for incontinence (open)	11	151	7	274	5	320	5	384	6	360
PSARP	9	32	8	55	8	65	6	93	5	78
Reversal of colostomy	8	56	7	80	7	101	3	242	3	313
Others	66	61	67	126	94	84	81	94	139	138
Total/mean	1,010	74	965	118	1,048	114	1,168	118	1,123	128

*Procedures reported by centers in the category “Others” included: Hernias, JJ-stent removal, varicocele, hydrocele, urogenital sinus correction, urachal cyst correction, labia minora hypertrophy correction, various biopsies, vesicostomy, ureterostomy reconstruction, and buried penis correction*.

To investigate whether the obtained waiting list data corresponded to the actual number of surgical procedures and outpatient consultations performed, before and during the study period, all centers were requested to further retrospectively report administrative data on these activities from January 2019 throughout September 2021. To compare with pre-COVID-19 capacities, as well as normal seasonal variation, this data was presented per annum. Moreover, to compare with the corresponding outpatient activities, data on external referrals for pediatric surgical evaluation during the same period was obtained from two participating centers.

Synchronously, qualitative online surveys regarding financial, logistic, and organizational changes were obtained using Redcap® (questions asked can be found in Figure 6). The participants were surveyed at four timepoints during the study period (June, October, January, and April, respectively).

### Statistics

Descriptive statistics were based on absolute numbers from reported waiting list at the five study timepoints. Given the explorative nature of our data, further advanced hypothesis testing models were not considered appropriate. Centers that were not able to provide complete waiting list data on the respective procedures for all timepoints were not included in the quantitative results.

Since data from large-volume centers might dominate the statistical tendencies in the general results, we assessed the proportional waiting list changes, to level out possible skewing from varying center sizes and operative volumes. The mean of the percentual waiting list changes from each center were assessed with reference to the study baseline (March 2020). Due to low sample-sizes and high variance in rare procedures, this was only deemed suitable for a limited number of high-volume procedures, defined in this study as >10 patients per center waiting on average at each timepoint (i.e., hypospadias, phimosis, non-descended testes, cystoscopy).

Numeric survey data is presented separately in percentages in stacked bar charts. Data and figures were analyzed using Microsoft® Office Excel 2016 and IBM® SPSS Statistics version 25.

## Results

A total of 10 tertiary European centers of pediatric urology, representing a background population of 21.6 million people (individually ranging from 1.2 to 3.3 million), were included prospectively in the study. According to 2019 cross European demographics, this corresponds to a total pediatric reference population <15 years of 3.26 million (9). All participating centers experienced a complete initial COVID-19 related closedown of elective pediatric urology cases, which happened medio March 2020 (effectuated between March 3^rd^ and 17^th^ and ended between March 20^th^ and September 1^st^, respectively).

### Quantitative Data

**Figure 1:**
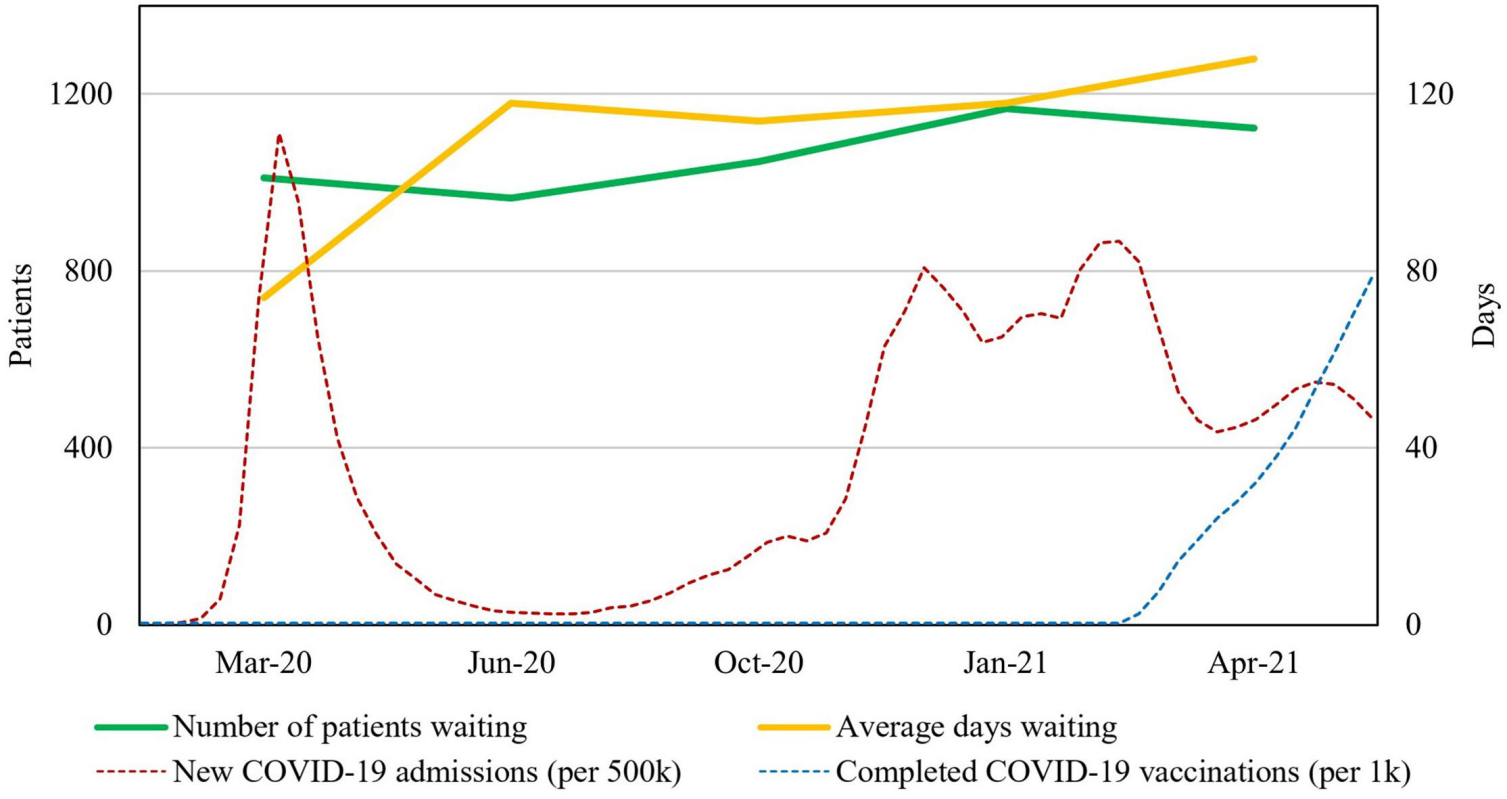
Waiting list tendencies during the COVID-19 pandemic. Total number of patients and average days on waiting lists across all participating centers for all procedures of interests are presented in absolute numbers. Mean cross-European data on new COVID-19 admissions and completed vaccinations are shown in absolute numbers for intuitive comparison (note these should be read from secondary Y-axis).

Seven centers (70%) were able to provide complete waiting list data for all timepoints, corresponding to a reference population of 17.7 million inhabitants. Results from the total counting of waiting lists at the five study timepoints across all participating centers are first presented in absolute numbers (Figure 1, Table 2). An increase in mean days waiting was seen during spring 2020 and again during late fall. This corresponded to the fluctuations of the first and second wave of infections in Europe. The total number of patients waiting for surgery did not increase on a broadscale during the first wave of infections, however it did increase during the second wave.

Both the number of patients and the mean days waiting generally decreased during the summer of 2020, after the first wave of infections, whereas a corresponding decrease was not seen after the second wave. At the end of the study, the included pediatric urology centers had increased the overall number of patients on waiting lists with 11% (from 1,010 to 1,123 patients), whereas mean days on waiting list had accumulated with 73% (from 74 to 128 days) (Figure 1). Over the entire course of the study period, the total accumulation of waiting days consequently attenuated with 6,102 days within the study population.

**Figure 2:**
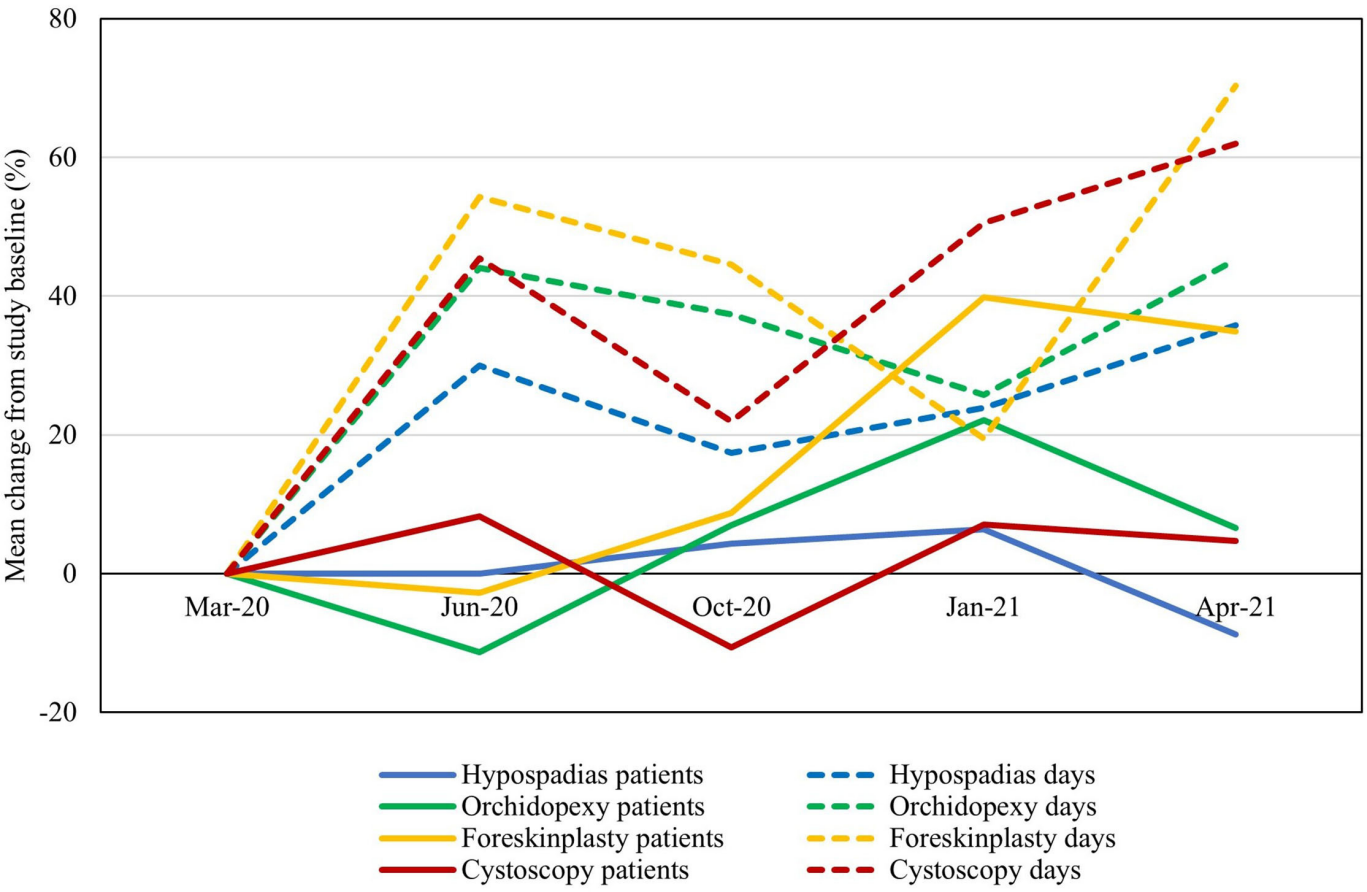
Proportional changes in number of patients (straight lines) and mean days (dotted lines) on waiting lists for high-volume pediatric urological procedures during the total study period. The mean proportional changes (in percentage) across all centers are presented with reference to study baseline (March 2020).

Evaluation of the proportional changes in waiting lists for high-volume procedures generally revealed clear gradual increase in the average days spent on waiting lists at the end of the study period (increasing with 62% for cystoscopy, 45% for orchidopexy, 36% for hypospadias and 70% for foreskinplasty). The changes in waiting lists corresponded to the nationwide fluctuations in the COVID-19 pandemic, although with some possible delay between new waves of infections and increasing waiting lists (Figure 2, Supplementary Table 1). The number of patients waiting remained stable until finally increasing in some procedures during the second half of the study period (final increase of 5% for cystoscopy, 7% for orchidopexy, −9% for hypospadias and 35% for foreskinplasty). These changes corresponded to a delay in seeing patients at the outpatient clinic for planning new surgical interventions.

**Figure 3:**
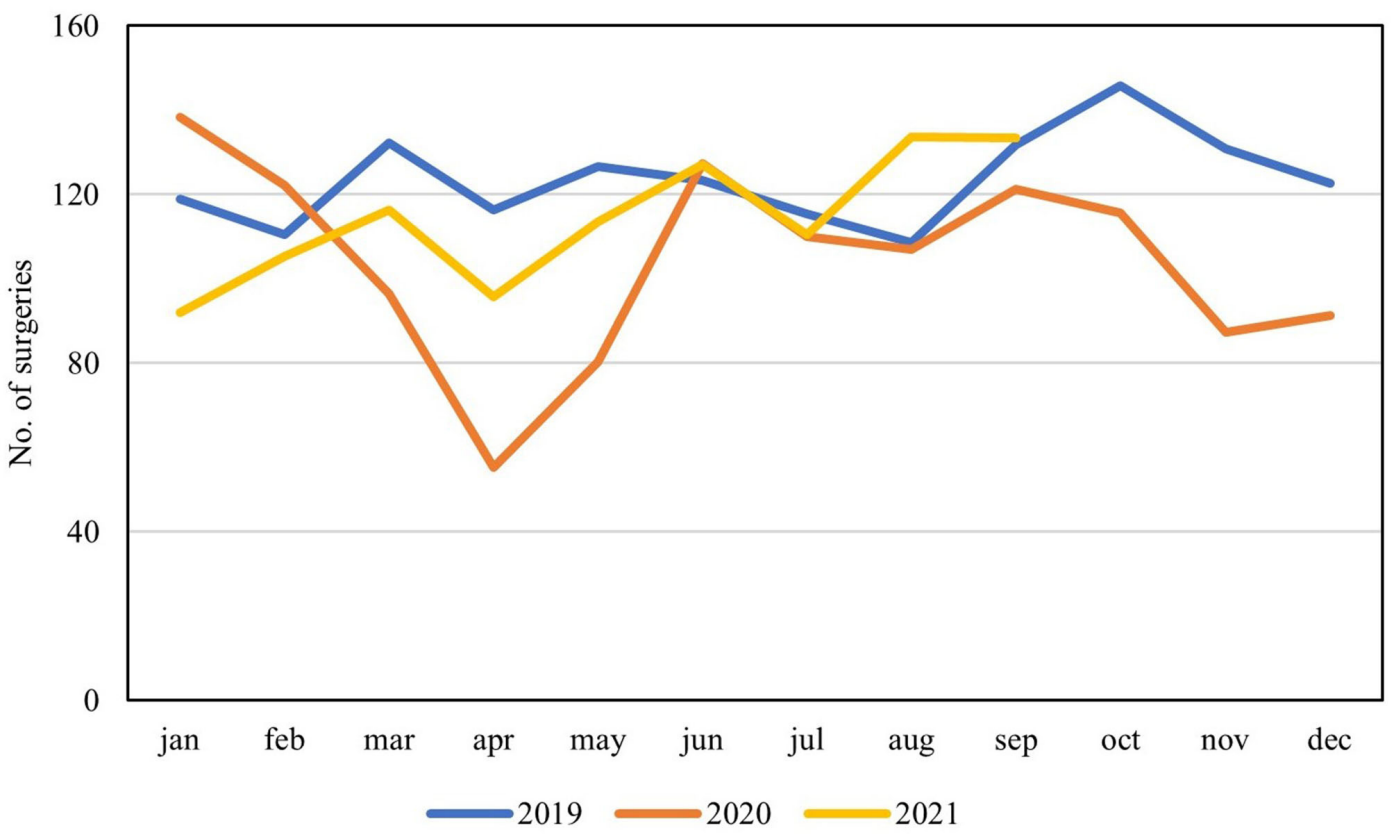
Mean monthly number of overall surgeries performed at seven participating centers during the past 3 years presented per annum in absolute numbers.

**Figure 4:**
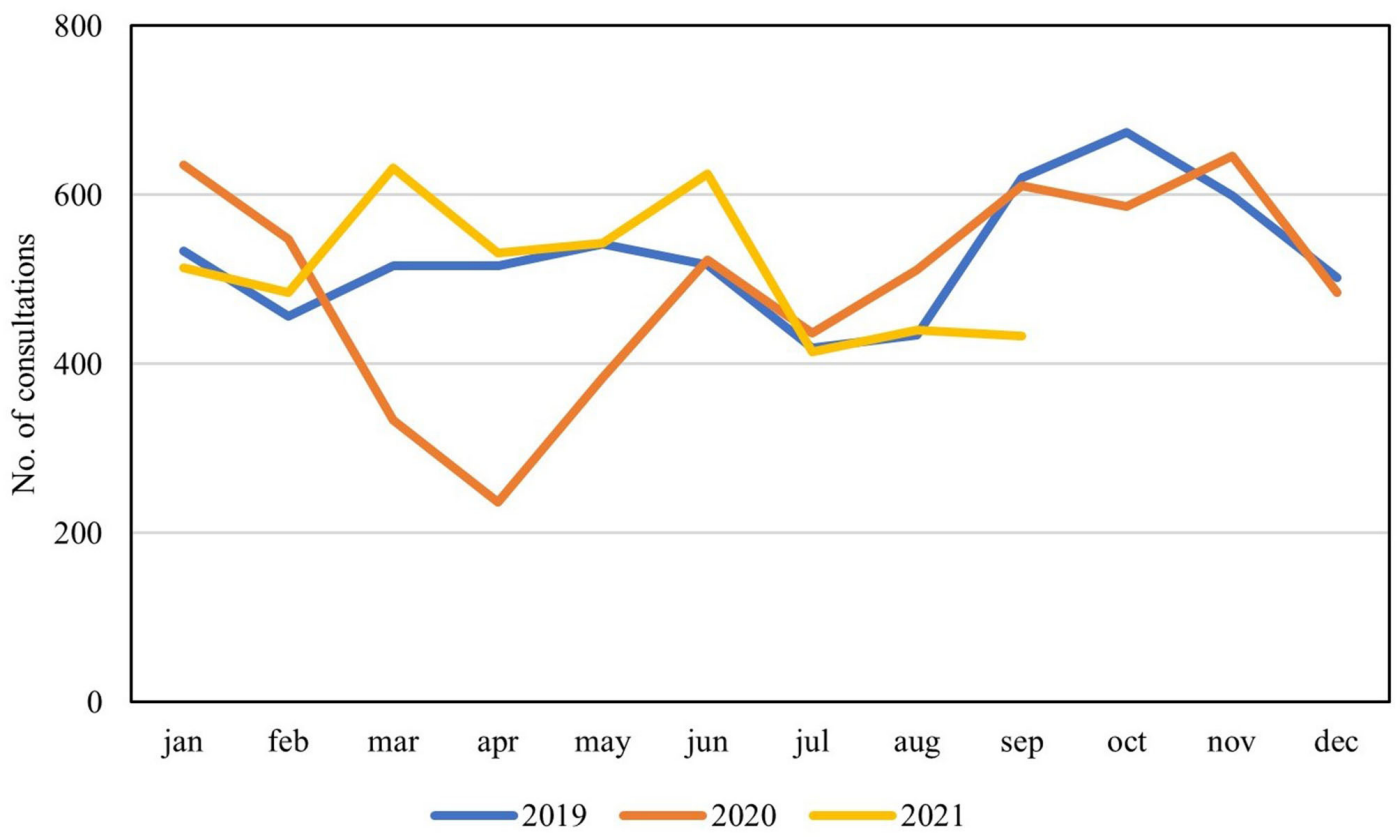
Mean monthly number of overall consultations performed at seven participating centers during the past 3 years presented per annum in absolute numbers.

**Figure 5:**
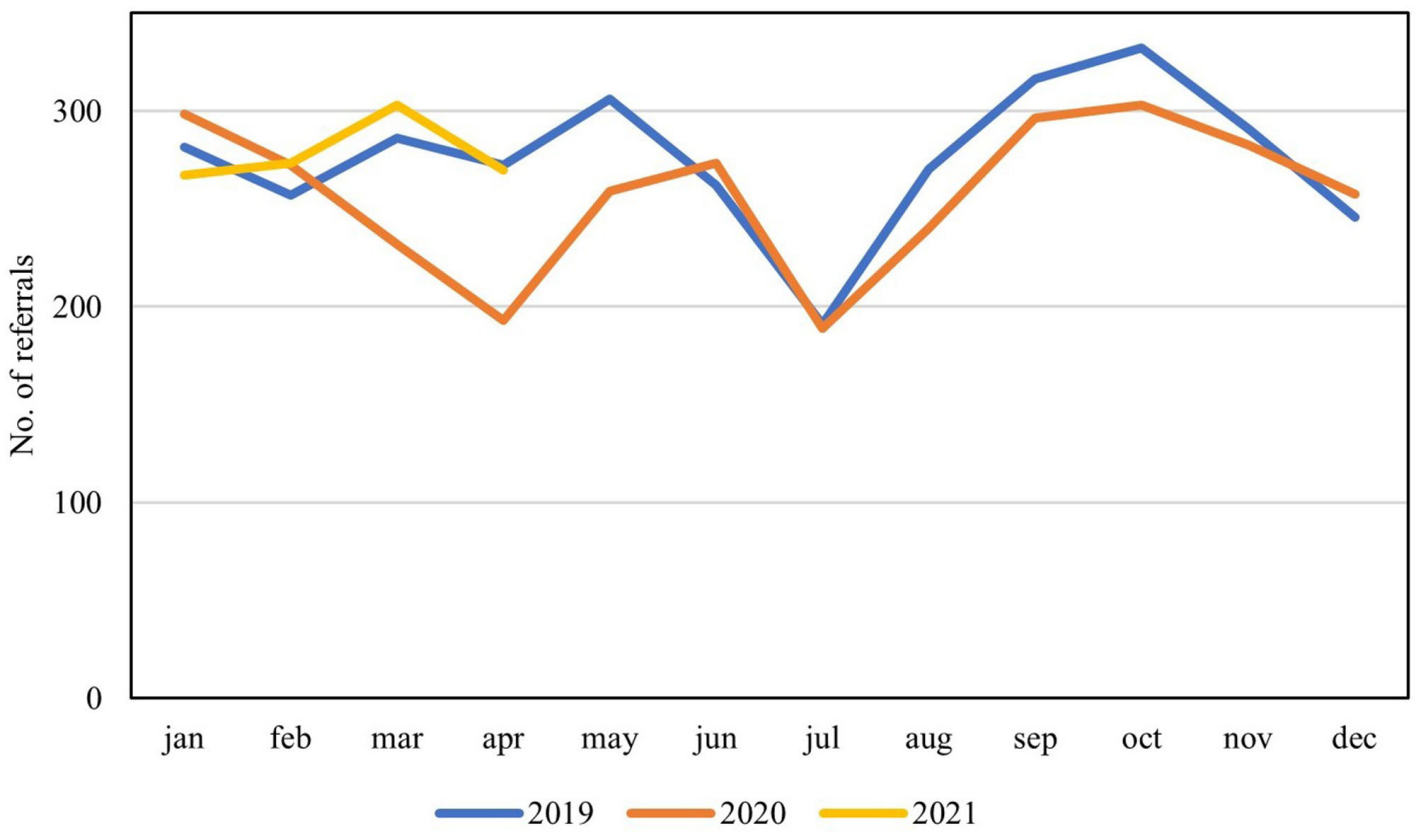
Mean number of overall external referrals to pediatric surgical evaluation received at two participating centers during the past 3 years presented per annum in absolute numbers.

Seven centers (70%) were able to provide additional administrative data on performed surgical procedures and outpatient consultations. Tendencies confirmed decreased activities with a clear dip during the first pandemic wave, when compared to activity levels of 2019, corresponding to a stagnation in number of patients entering the waiting lists during the first part of the study (Figures 3, 4). When evaluating external referrals, a corresponding decrease in activity was also seen during spring 2020 (Figure 5).

### Qualitative Data

**Figure 6:**
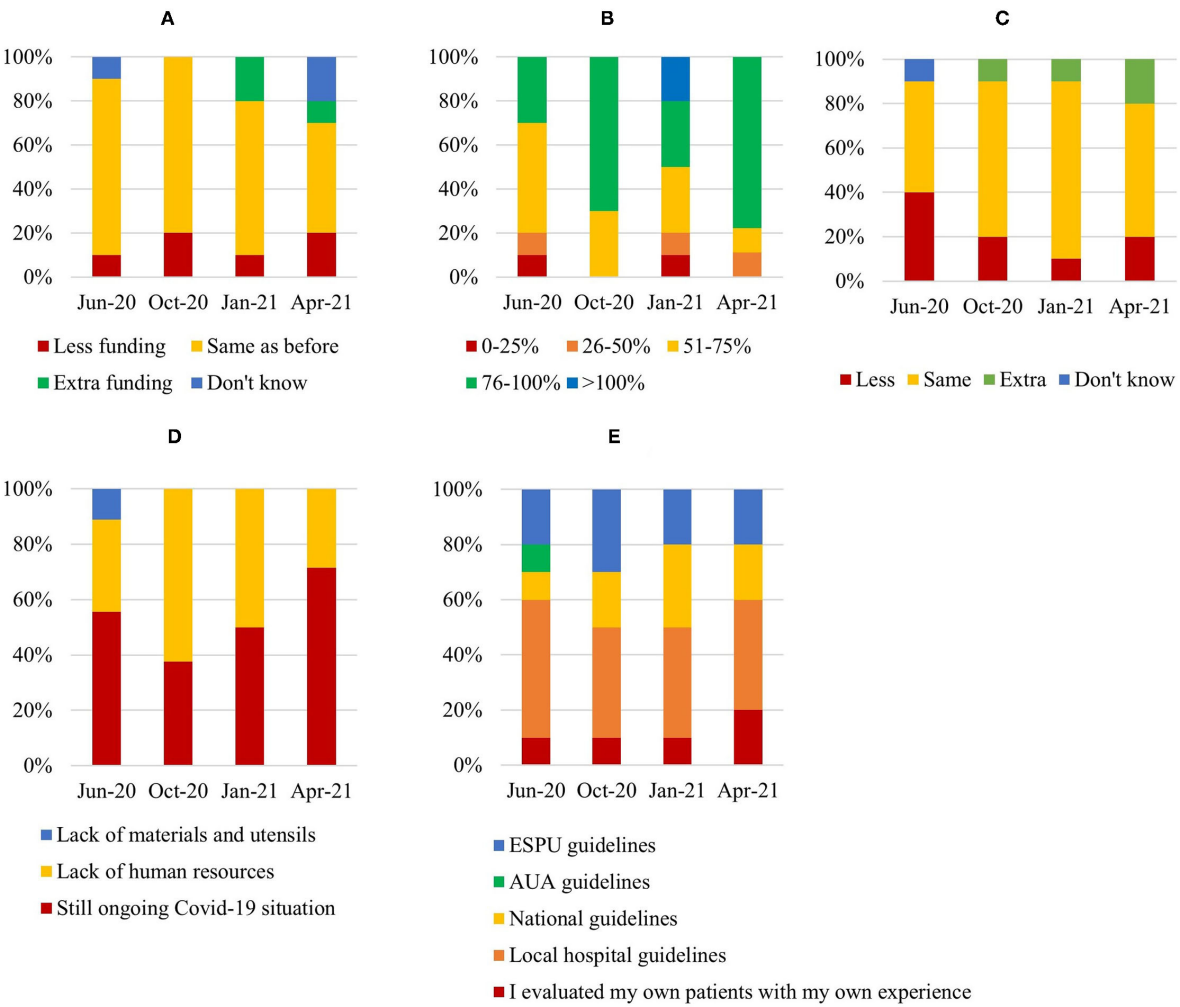
Compiled survey data from all centers. Each subfigure represents a question from the survey: **(A)** Have your financial resources changed after the Covid-19 close-down? **(B)** Have you now reached the same OR capacity as before close-down? **(C)** Have you been able to increase team-member working hours (i.e., surgeons, anesthesiologists, OR nurses and postop team), in order to decrease the waiting list? **(D)** What is the main limiting factor right now for full OR capacity? **(E)** Did you follow ESPU or national guidelines to deal with the waiting list during and after closedown?

All 10 centers provided full survey data from each of the four survey timepoints. Financial resources remained unreduced in ~80% of the centers during the study period but with uncertainties at the last survey. A tendency of extra funding was reported in 15% of centers during the second half of the study (Figure 6A). A tendency of lesser funding was reported in an equal number of centers (15%) and these centers were also more heavily burdened by the COVID-19 pandemics at a national level. Operating room (OR) capacity was reduced with at least 75% at 3-months after the closedown in 70% of centers, while 30% had been able to re-establish their capacity to 76–100% of normal. In approximately half of the centers a decrease in OR capacity clearly followed the first and second wave of infections in Europe (during the spring and fall of 2020, respectively) (Figure 6B). Three months after initial closedown (June 2020) 40% experienced a decrease in OR staff compared to normally, however during the remaining study period, an increasing number of centers were able to sustain or even increase the OR staff working hours to endure the increasing caseload (Figure 6C). Consequently, rather than lack of human or material resources, at the end of the study period, general ongoing COVID-19 situation was viewed as the main limiting factor for OR capacity throughout the study period in most centers (Figure 6D).

In January 2021, 60% of centers reported a second closedown (effectuated between October 5th and December 2^nd^ and lasting on average 2–3 months, although still ongoing in two centers at the end of the study period) with a corresponding decrease in OR capacity in almost 50% of centers. Management of waiting lists was done in accordance with various guidelines depending on each center, with ~20% of centers following ESPU guidelines and 40% following local hospital guidelines, and centers generally adhered to the same guidelines throughout the study period (Figure 6E).

### Combined Quantitative and Qualitative Data

**Figure 7:**
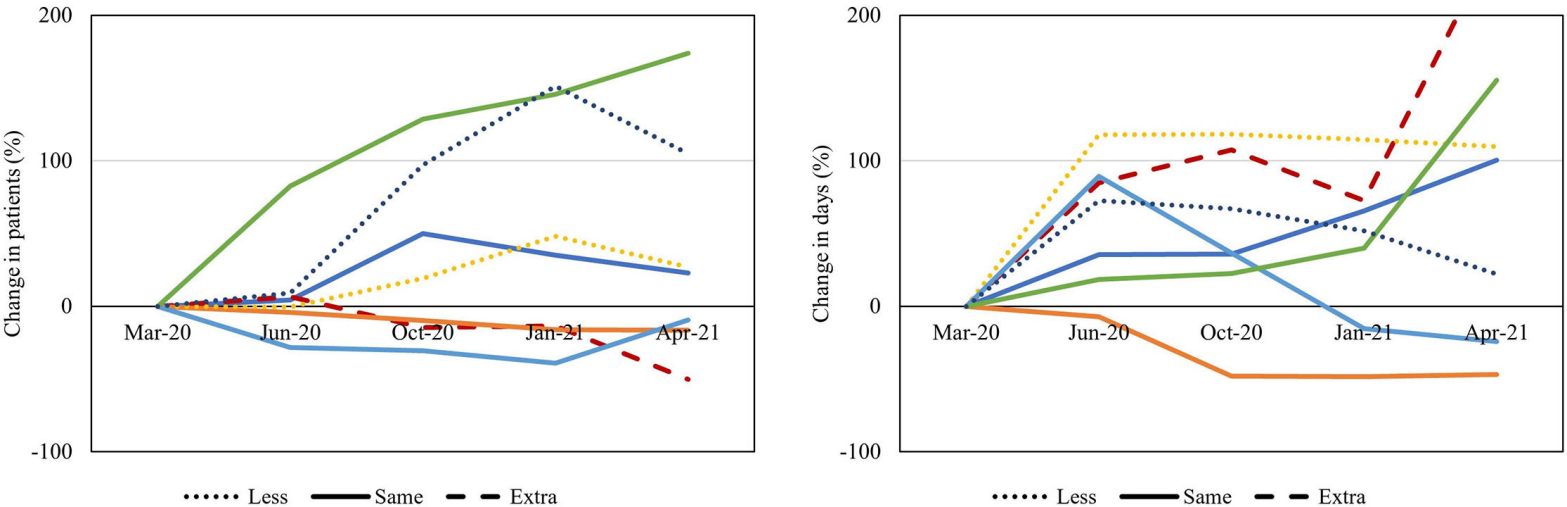
Funding during the study period. Proportional changes in number of patients and mean days waiting for the mean of all high-volume procedures presented in percentage for each center (represented in colors) in relation to funding status at the end of the study (dotted, dashed and straight lines, respectively).

**Figure 8:**
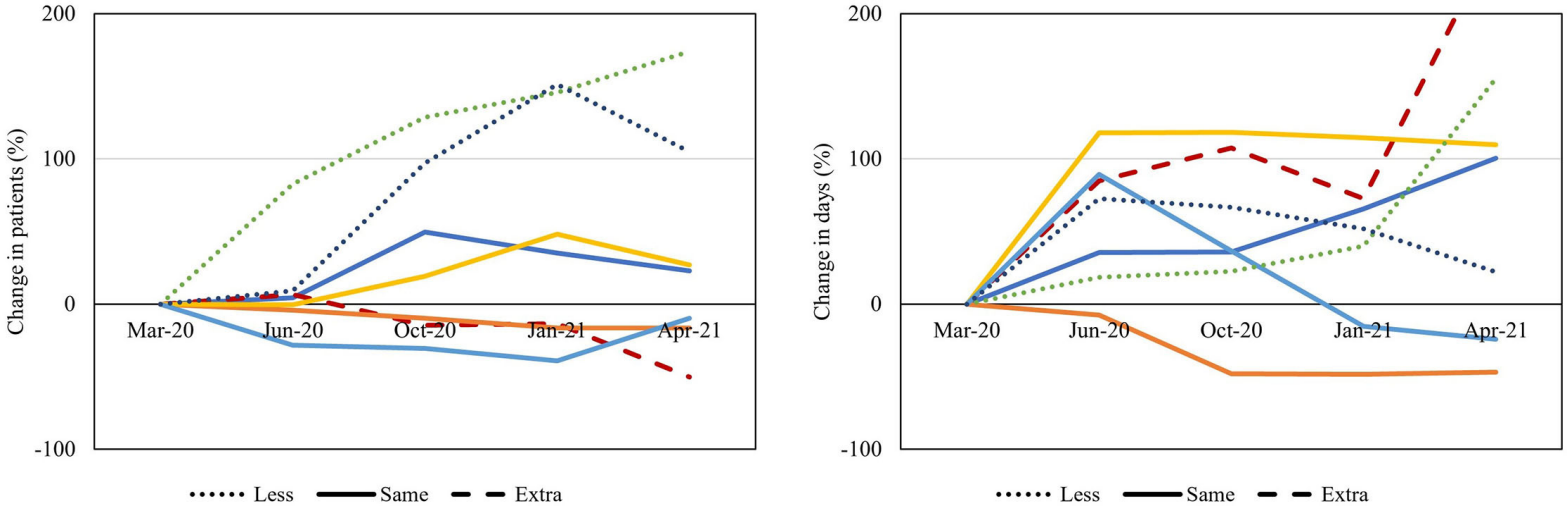
Ability to increase operating room staff working hours. Proportional changes in number of patients and mean days waiting for the mean of all high-volume procedures presented in percentage for each center (represented in colors) in relation to staff working hours at the end of the study (dotted, dashed and straight lines, respectively).

When evaluating the mean proportional changes for all high-volume procedures in relation to funding, a larger increase in number of patients waiting was seen in centers reporting on less funding in the final survey compared to centers reporting unreduced or additional funding. This tendency was not seen in relation to mean days waiting (Figure 7). Similar tendencies were seen when evaluating the proportional changes in relation to OR staff working hours; less ability to increase staff working hours at the end of the study was correlated with increased numbers of patients waiting, whereas this tendency was not seen in relation to mean number of days waiting (Figure 8).

## Discussion

This European multicenter study is the first of its kind to report on prospectively gathered multicenter real-time data on the ongoing impacts of COVID-19 on elective surgeries in European pediatric urological populations. Our study presents waiting list outcomes for a variety of 17 urological procedures from tertiary European referral centers for pediatric urology within the first year of the COVID-19 pandemic, covering a population of ~22 million persons.

Since the outbreak of the COVID-19 pandemic, pediatric surgical activities has evidently been reduced worldwide (10–13) and although the quality of the surgical treatment does not seem to have diminished during the pandemic (14), increased time to surgery has been documented in various urgent pediatric cases, such as acute appendicitis (15, 16) and testicular torsion (17, 18). Further, the pandemic has impacted on the training of new pediatric surgeons (19) and presumably also on non-COVID-19 related pediatric research (20). In non-urgent elective cases however, the effect of the current administrative prioritizations, halting surgical activities, has not yet been investigated prospectively on a multicenter level.

In our material we found that during the first half of the study period, the number of patients on waiting lists for most surgeries remained stable and did not increase until the second half. This is explained by a reduced intake *via* outpatient clinics, consequently halting new OR bookings during the initial closedown.

Later during the study period, the number of patients on waiting lists and accumulated waiting time increased for most procedures, although we did encounter tendencies that some procedures were prioritized. These procedures, although many of them performed on rare diseases with consequently small sample-sizes, often represented cases with life- or organ-threatening conditions (pyeloplasty, bladder exstrophy, heminephrectomy). Still, for now, any conclusions regarding low-volume procedures can only be speculative. However, when evaluating the proportional waiting list changes at the end of the study period, we did find a clear increase in the accumulated time waiting for all four high-volume surgeries (hypospadias, cystoscopy, foreskinplasty and orchidopexy), in addition to an increase in number of patients waiting.

Attenuated waiting lists generally reflected the first and second wave of European COVID-19 infections, and indications of compensatory activities to decrease waiting lists were seen after the first wave, during summer 2020. However, similar tendencies were not found after the second wave, where waiting list continued to increase, which may indicate an exhaustion of the center capacities, limiting the ability to compensate for attenuated waiting lists with more elective operating room time. This exposes a limit of robustness in the health care systems with regards to compensatory means that might have reached an upper limit in many centers at that time. Although the data material obtained in this study was largely explorative, allowing only for descriptive statistical analysis, the gradually attenuating waiting lists during the first year of COVID-19 were convincing, and in general corresponded to similar reports from adult surgery waiting lists in the same period (21–24).

According to the survey data, OR capacity decreased in more than half of the participating centers, however, most centers were able to maintain or increase their financial budget (60–80%), as well as OR staffing (70%), during the second half of the study period. Still, at the end of the study, half of the centers still reported an OR capacity below 75%, due to an ongoing COVID-19 situation. Guideline adherence for prioritizing surgical cases, varied greatly within the study group, however, remained unchanged throughout the study period. Our findings related to high-volume cases support that prioritization was quite similar between centers, although different guidelines were used.

Not all centers reported on a 2nd closedown (60%), but waiting lists were markedly increased at the second wave, much more than after the first. This may be due to closing of elective surgeries but still performing the outpatient clinics. One of the most critical measures of the quality of a country's health care system is how long patients must wait to access medical care. Our study demonstrated a waiting time of ~2.5 months for a surgical intervention in general anesthesia before the pandemic, to more than 4 months for the average pediatric patient after 1 year of the pandemic.

Patients perspectives have been reported to play an important role in the rate of hospital contacts during the pandemic, and although this was not possible for us to evaluate in the current study, we do suspect that fear of seeking healthcare, as seen in other studies on emergency conditions, has also influenced elective waiting lists (12, 15, 17, 18, 25, 26). Several new advances have been implemented in many clinics during the pandemic, to meet with the emerging challenges. Telemedicine for one has proven to be a valuable tool in the reduction of physical outpatient activity by replacing actual meetings with either telephone, video or online consultations. Surveys have demonstrated acceptable user satisfaction rates despite short-notice introduction of the new technique (27, 28), although possibly susceptible to social and economic disparities (29). New telemedical guidelines have been proposed and might very well find a more permanent place in future pediatric practices (30).

European centers will ongoingly need to plan more elective surgeries to address the currently increased caseload. This study should be understood in the context of an ongoing worldwide health crisis from which the full impact on healthcare systems are yet to be seen, warranting further monitoring and analysis. In respect to waiting time for interventions in patients which we know have morbidity in delay for treatment, we do not have tools for estimating loss of quality of life, and neither cost related to postponing surgeries (i.e., medications, monitoring, outpatient visits, parents needing to stay home from work to take care of their child, children not attending school). It is feasible, however, that the impact of patients and parents staying home longer, while waiting for surgery, will have social, educational and economic consequences for the families, as well as possible progression of the underlying medical conditions (31).

At present, some European countries are still facing a heavy burden on healthcare due to the COVID-19 situation. In addition, we do not know how the European health care systems will react during a post-COVID-19 period, in the sense that health care workers have been exhausted by the emergency of high workload and long working hours. In addition, patients that are subjected to post-COVID-19 symptoms may cause further challenges to the healthcare systems to provide and prioritize care of patients (32, 33). By these means, pediatric urology waiting lists may further increase before it can go back to the same level as before COVID-19.

The prospective study design ensured that data was gathered consistently throughout the study period. By including a variety of centers throughout Europe, we believe that the waiting list tendencies found among the participating centers can indeed be translated to other countries and other regions or continents with similar socio-economic health resources. If looking at the whole population of the European Union (447 million), and if our data provided could represent the whole union, then an extrapolation with a 30-fold increment would represent the whole union; waiting lists could be expected to increase with 183 060 accumulated waiting days due to the pandemic (3 390 new pediatric patients waiting and an increased waiting time of 54 days).

In conclusion, closure of elective interventions affected the pediatric urology population by an increased number of patients waiting for surgery and an increase in time on waiting lists (70% longer) for surgical interventions. Some centers dealt with the problem by increasing their operating resources off-hours, however, it is still not known how many more patients will suffer from delays before it turns back to normal again. Political decision-making and unified forces will be needed to restore a reliable health care system that attends the pediatric population in a timely manner.

## Data Availability Statement

The original contributions presented in the study are included in the article/Supplementary Material, further inquiries can be directed to the corresponding author.

## Ethics Statement

Ethical review and approval was not required for the study on human participants in accordance with the local legislation and institutional requirements. Written informed consent from the participants' legal guardian/next of kin was not required to participate in this study in accordance with the national legislation and the institutional requirements.

## Author Contributions

NJ, MF, AC, and NK developed the study design. NJ and MF analyzed the data and drafted the manuscript. All authors contributed with study data from their own center and critically reviewed and accepted the final manuscript.

## Funding

NJ and MF has received unrestricted research grants from the Novo Nordisk Foundation (NNFSA170030576).

## Conflict of Interest

The authors declare that the research was conducted in the absence of any commercial or financial relationships that could be construed as a potential conflict of interest.

## Publisher's Note

All claims expressed in this article are solely those of the authors and do not necessarily represent those of their affiliated organizations, or those of the publisher, the editors and the reviewers. Any product that may be evaluated in this article, or claim that may be made by its manufacturer, is not guaranteed or endorsed by the publisher.

## Abbreviations

AUA: American Urology Association
ESPU: European Society of Pediatric Urology
ERN: European Reference Network
eUROGEN: ERN for rare urogenital diseases and complex conditions
OR: Operating room.

## References

[1] Recommendations from the EAU/ESPU Paediatric Urology Guidelines Panel applicable during the COVID-19 pandemic Diagnosis and outpatient clinics for paediatric urology cases. Available online at: https://www.espu.org/members/publications/380-changes-in-paediatric-urology-practice-in-the-context-of-the-covid-19-outbreak

[2] Tur AB Prieto JC Gómez-Fraile A Corbetta JP. The effect of the Covid-19 Pandemic on pediatric urology. Int Braz J Urol. (2020) 46:133–44. 10.1590/s1677-5538.ibju.2020.s112 32568499 PMC7719986

[3] Campi R Tellini R Grosso AA Pecoraro A Mari A Raspollini MR. Exploring the diversity and predictors of histopathological findings across the European association of urology guidelines office rapid reaction group priority groups for patients with renal tumors: implications for individualized prioritization of renal cancer care. Eur Urol Open Sci. (2021) 34:5–9. 10.1016/j.euros.2021.09.009 34761237 PMC8567362

[4] Esposito C Masieri L Castagnetti M Crocetto F Escolino M. Letter to the editor: robot-assisted and minimally invasive pediatric surgery and urology during the COVID-19 pandemic: a short literature review. J Laparoendosc Adv Surg Tech. (2020) 30:915–8. 10.1089/lap.2020.0251 32498612

[5] Cini C Bortot G Sforza S Mantovani A Landi L Esposito C. Paediatric urology practice during COVID-19 pandemic. J Pediatr Urol. (2020) 16:295–6. 10.1016/j.jpurol.2020.04.023 32359910 PMC7195101

[6] Fernandez N Caicedo JI. Impact of COVID-19 on the future of pediatric urology practice. Do guidelines apply to medical practice worldwide? J Pediatr Urol. (2020) 16:291–2. 10.1016/j.jpurol.2020.05.001 32467025 PMC7240259

[7] Implementing decision containing criteria for establishing and evaluating ERNs including including the exchange and dissemination of information about the ERNs. Available online at: https://ec.europa.eu/health/publications/implementing-decision-containing-criteria-establishing-and-evaluating-erns-including-exchange-and_en (accessed Febraury 26, 2022).

[8] Publications & Data | European Centre for Disease Prevention and Control. Available online at: https://www.ecdc.europa.eu/en/publications-data (accessed June 10, 2021).

[9] Population structure and ageing - Statistics Explained. Available online at: https://ec.europa.eu/eurostat/statistics-explained/index.php?title=Population_structure_and_ageing (accessed Febraury 25, 2022).

[10] Garriboli M Mishra P Taghizadeh A Paul A. The response of a tertiary paediatric urology unit to the COVID-19 Pandemic in central London: what have we learned? Br J Surg. (2020) 107:e578–80. 10.1002/bjs.11978 32924140 PMC7929335

[11] Gunadi Idham Y Paramita VMW Fauzi AR Dwihantoro A Makhmudi A. The Impact of COVID-19 pandemic on pediatric surgery practice: a cross-sectional study. Ann Med Surg. (2020) 59:96–100. 10.1016/j.amsu.2020.09.020 32953094 PMC7491416

[12] Farooq MA Al Kabir SMH Chowdhury TK Sadia A Alam MA Farhad T. Changes in children's surgical services during the COVID-19 pandemic at a tertiary-level government hospital in a lower middle-income country. BMJ Paediatr Open. (2021) 5:e001066. 10.1136/bmjpo-2021-001066 34192202 PMC8015790

[13] Wei Y Yu C Zhao TX Lin T Dawei HE Wu S de. The impact of the COVID-19 pandemic on pediatric operations: a retrospective study of Chinese children. Ital J Pediatr. (2020) 46:155. 10.1186/s13052-020-00915-3 33066803 PMC7563908

[14] Merino-MateoLTordable OjedaCCabezalí BarbanchoDGómez FraileA. Impact of the COVID-19 pandemic on the surgical activity of Pediatric Urology: analysis of postoperative complications according to the Clavien-Dindo classification. Actas Urol Esp. (2020) 44:659–64. 10.1016/j.acuro.2020.09.00333069488PMC7498256

[15] Schäfer F-M Meyer J Kellnar S Warmbrunn J Schuster T Simon S. Increased incidence of perforated appendicitis in children during COVID-19 pandemic in a bavarian multi-center study. Front Pediatr. (2021) 9:683607. 10.3389/fped.2021.683607 34026695 PMC8138624

[16] Montalva L Haffreingue A Ali L Clariot S Julien-Marsollier F Ghoneimi A El. The role of a pediatric tertiary care center in avoiding collateral damage for children with acute appendicitis during the COVID-19 outbreak. Pediatr Surg Int. (2020) 36:1397–405. 10.1007/s00383-020-04759-0 33070203 PMC7568762

[17] Holzman SA Ahn JJ Baker Z Chuang K-W Copp HL Davidson J. A multicenter study of acute testicular torsion in the time of COVID-19. J Pediatr Urol. (2021). 10.1016/j.jpurol.2021.03.013 33832873 PMC7977032

[18] Pogorelić Z Milanović K Veršić AB Pasini M Divković D Pavlović O. Is there an increased incidence of orchiectomy in pediatric patients with acute testicular torsion during COVID-19 pandemic?–A retrospective multicenter study. J Pediatr Urol. (2021) 17: 479.e1–6. 10.1016/j.jpurol.2021.04.017 33994321 PMC8087574

[19] Mariani A Tiryaki S Harms M Orlov V Enache T Bidault-Jourdainne V. What we learned from the Covid-19 first wave: a survey from Young Pediatric Urology Committee (YPUC) from ESPU. Minerva Pediatr. (2021). 10.23736/S2724-5276.21.06325-4. [Epub ahead of print]. 33949828

[20] Harper L Kalfa N Beckers GMA Kaefer M Nieuwhof-Leppink AJ Fossum M. The impact of COVID-19 on research. J Pediatr Urol. (2020) 16:715–6. 10.1016/j.jpurol.2020.07.002 32713792 PMC7343645

[21] Ashfaq A Gray GM Carapellucci J Amankwah EK Ahumada LM Rehman M. Impact of coronavirus-2019 on pediatric and adult heart transplantation waitlist activity and mortality in the United States: a descriptive approach. Lancet Reg Heal Am. (2021) 3:100060. 10.1016/j.lana.2021.100060 34786570 PMC8581289

[22] Muschol J Gissel C. COVID-19 pandemic and waiting times in outpatient specialist care in Germany: an empirical analysis. BMC Health Serv Res. (2021) 21:1076. 10.1186/s12913-021-07094-9 34635091 PMC8503703

[23] García-Rojo E Manfredi C Santos-Pérez-de-la-Blanca R Tejido-Sánchez Á García-Gómez B Aliaga-Benítez M. [Impact of COVID-19 outbreak on urology surgical waiting lists and waiting lists prioritization strategies in the Post-COVID-19 era]. Actas Urol Esp. (2021) 45:207–14. 10.1016/j.acuro.2020.11.001 33546905

[24] Uimonen M Kuitunen I Paloneva J Launonen AP Ponkilainen V Mattila VM. The impact of the COVID-19 pandemic on waiting times for elective surgery patients: a multicenter study. PLoS ONE. (2021) 16:e0253875. 10.1371/journal.pone.0253875 34228727 PMC8259989

[25] AmparoreDCampiRCheccucciEPianaASicaMGrossoAA. Patients' perspective on the use of telemedicine for outpatient urological visits: Learning from the COVID-19 outbreak. Actas Urol Esp. (2020) 44:637–8. 10.1016/j.acuro.2020.06.00832843151PMC7359779

[26] Campi R Tellini R Grosso AA Amparore D Mari A Viola L. Deferring elective urologic surgery during the COVID-19 pandemic: the patients' perspective. Urology. (2021) 147:21–6. 10.1016/j.urology.2020.09.015 32979378 PMC7513799

[27] Gan Z Lee SY Weiss DA Van Batavia J Siu S Frazier J. Single institution experience with telemedicine for pediatric urology outpatient visits: adapting to COVID-19 restrictions, patient satisfaction, and future utilization. J Pediatr Urol. (2021) 17:480.e1-480.e7. 10.1016/j.jpurol.2021.05.012 34078574 PMC8491551

[28] Schmidtberg LC Grindle C Hersh DS Rowe C Healy J Hughes CD. Telehealth in pediatric surgical subspecialties: rapid adoption in the setting of COVID-19. Telemed e-Health. (2021) 28:344–52. 10.1089/tmj.2021.0080 34101508

[29] Winkelman AJ Beller HL Morgan KE Corbett ST Leroy SV Noona SW. Benefits and barriers to pediatric tele-urology during the COVID-19 pandemic. J Pediatr Urol. (2020) 16:840.e1–6. 10.1016/j.jpurol.2020.09.028 33077389 PMC7543732

[30] Charnell AM Hoen L't Sforza S Spinoit AF Radford A. Remote consultations in paediatric urology–Not just for pandemics? J Pediatr Urol. (2021) 17:260–2. 10.1016/j.jpurol.2021.01.001 33478900

[31] Forner D Leslie PK Aldaihani A Bezuhly M Noel CW Horne D. Psychosocial distress in parents with children awaiting surgery during the COVID-19 pandemic. Child. (2022) 9:87. 10.3390/children9010087 35053712 PMC8774209

[32] Saloner B Parish K Julie Ward MA Grace DiLaura R Sharon Dolovich J. Persistent symptoms in patients after acute COVID-19. JAMA. (2020) 324:603–5. 10.1001/jama.2020.12603 32644129 PMC7349096

[33] Tenforde MW Kim SS Lindsell CJ Billig Rose E Shapiro NI Files DC. Symptom duration and risk factors for delayed return to usual health among outpatients with covid-19 in a multistate health care systems network—United States, March–June 2020. Morb Mortal Wkly Rep. (2020) 69:993–8. 10.15585/mmwr.mm6930e1 32730238 PMC7392393

