# Supplementary material for: How the First Year of COVID-19 Affected Elective Pediatric Urology Patients: A Longitudinal Study Based on Waiting Lists and Surveys From 10 European Centers
Source: Front Public Health. 2022 Apr 28;10:874758. doi: 10.3389/fpubh.2022.874758 (PMC9096088; doi:10.3389/fpubh.2022.874758)
Supplement: Supplementary file 1 [file Table_1.DOCX]

Supplementary table 1. High-volume procedures per center and in total for each study timepoint. Mean proportional changes in number of patients and mean days on waiting lists since study baseline (March 2020) are presented in percentages.

|  | **Patients on waiting list** | | | | | **Mean days waiting** | | |  |  | **Change in patients waiting from T0 (%)** | | | | | **Change in time waiting from T0 (%)** | | | | |
| --- | --- | --- | --- | --- | --- | --- | --- | --- | --- | --- | --- | --- | --- | --- | --- | --- | --- | --- | --- | --- |
| **Hypospadias repair** | T0 | T1 | T2 | T3 | T4 | T0 | T1 | T2 | T3 | T4 | T0 | T1 | T2 | T3 | T4 | T0 | T1 | T2 | T3 | T4 |
| Center 1 | 54 | 55 | 54 | 60 | 43 | 195 | 282 | 262 | 263 | 330 | 0 | 2 | 0 | 11 | -20 | 0 | 45 | 35 | 35 | 70 |
| Center 2 | 49 | 53 | 61 | 41 | 44 | 175 | 225 | 211 | 150 | 162 | 0 | 8 | 24 | -16 | -10 | 0 | 28 | 20 | -14 | -8 |
| Center 3 | 28 | 29 | 20 | 22 | 28 | 220 | 191 | 93 | 120 | 131 | 0 | 4 | -29 | -21 | 0 | 0 | -13 | -58 | -46 | -40 |
| Center 4 | 42 | 36 | 43 | 44 | 37 | 70 | 149 | 118 | 131 | 152 | 0 | -14 | 2 | 5 | -12 | 0 | 112 | 67 | 87 | 116 |
| Center 5 | 45 | 40 | 36 | 40 | 42 | 83 | 129 | 89 | 73 | 63 | 0 | -11 | -20 | -11 | -7 | 0 | 55 | 7 | -12 | -24 |
| Center 6 | 5 | 8 | 12 | 22 | 29 | 31 | 19 | 30 | 89 | 130 | 0 | 60 | 140 | 340 | 480 | 0 | -40 | -2 | 189 | 319 |
| Center 7 | 75 | 77 | 85 | 88 | 49 | 219 | 299 | 364 | 404 | 381 | 0 | 3 | 13 | 17 | -35 | 0 | 36 | 66 | 84 | 74 |
| Total / mean | 298 | 298 | 311 | 317 | 272 | 142 | 185 | 167 | 176 | 193 | 0 | 0 | 4 | 6 | -9 | 0 | 30 | 17 | 24 | 36 |
| **Orchidopexy** |  |  |  |  |  |  |  |  |  |  |  |  |  |  |  |  |  |  |  |  |
| Center 1 | 85 | 92 | 93 | 124 | 98 | 151 | 226 | 237 | 226 | 293 | 0 | 8 | 9 | 46 | 15 | 0 | 50 | 58 | 50 | 94 |
| Center 2 | 50 | 26 | 17 | 30 | 11 | 47 | 86 | 95 | 79 | 114 | 0 | -48 | -66 | -40 | -78 | 0 | 83 | 102 | 68 | 142 |
| Center 3 | 28 | 26 | 28 | 29 | 35 | 101 | 93 | 68 | 82 | 85 | 0 | -7 | 0 | 4 | 25 | 0 | -8 | -33 | -19 | -16 |
| Center 4 | 16 | 19 | 30 | 28 | 24 | 52 | 128 | 142 | 159 | 117 | 0 | 19 | 88 | 75 | 50 | 0 | 145 | 172 | 205 | 124 |
| Center 5 | 26 | 18 | 16 | 8 | 18 | 66 | 110 | 108 | 43 | 50 | 0 | -31 | -38 | -69 | -31 | 0 | 66 | 63 | -36 | -24 |
| Center 6 | 21 | 17 | 15 | 12 | 10 | 31 | 21 | 26 | 25 | 19 | 0 | -19 | -29 | -43 | -52 | 0 | -32 | -16 | -18 | -38 |
| Center 7 | 31 | 30 | 76 | 83 | 78 | 80 | 98 | 50 | 51 | 89 | 0 | -3 | 145 | 168 | 152 | 0 | 22 | -38 | -36 | 11 |
| Total / mean | 257 | 228 | 275 | 314 | 274 | 75 | 109 | 104 | 95 | 110 | 0 | -11 | 7 | 22 | 7 | 0 | 44 | 37 | 26 | 45 |
| **Foreskinplasty** |  |  |  |  |  |  |  |  |  |  |  |  |  |  |  |  |  |  |  |  |
| Center 1 | 65 | 70 | 80 | 98 | 106 | 163 | 236 | 261 | 274 | 299 | 0 | 8 | 23 | 51 | 63 | 0 | 44 | 60 | 68 | 83 |
| Center 2 | 17 | 24 | 21 | 19 | 2 | 27 | 56 | 90 | 94 | 255 | 0 | 41 | 24 | 12 | -88 | 0 | 107 | 230 | 246 | 839 |
| Center 3 | 8 | 6 | 11 | 6 | 4 | 158 | 131 | 53 | 26 | 34 | 0 | -25 | 38 | -25 | -50 | 0 | -17 | -66 | -84 | -79 |
| Center 4 | 7 | 7 | 7 | 13 | 10 | 68 | 169 | 178 | 133 | 124 | 0 | 0 | 0 | 86 | 43 | 0 | 148 | 162 | 95 | 82 |
| Center 5 | 28 | 15 | 12 | 12 | 19 | 44 | 112 | 76 | 46 | 35 | 0 | -46 | -57 | -57 | -32 | 0 | 153 | 72 | 3 | -21 |
| Center 6 | 28 | 25 | 29 | 52 | 75 | 32 | 35 | 31 | 8 | 115 | 0 | -11 | 4 | 86 | 168 | 0 | 7 | -3 | -75 | 254 |
| Center 7 | 30 | 31 | 39 | 56 | 31 | 69 | 129 | 123 | 91 | 95 | 0 | 3 | 30 | 87 | 3 | 0 | 88 | 80 | 33 | 39 |
| Total / mean | 183 | 178 | 199 | 256 | 247 | 80 | 124 | 116 | 96 | 137 | 0 | -3 | 9 | 40 | 35 | 0 | 54 | 45 | 19 | 70 |
| **Cystoscopy** |  |  |  |  |  |  |  |  |  |  |  |  |  |  |  |  |  |  |  |  |
| Center 1 | 3 | 3 | 8 | 4 | 4 | 199 | 206 | 184 | 418 | 508 | 0 | 0 | 167 | 33 | 33 | 0 | 3 | -8 | 110 | 155 |
| Center 2 | 20 | 25 | 12 | 18 | 15 | 33 | 73 | 59 | 30 | 45 | 0 | 25 | -40 | -10 | -25 | 0 | 121 | 78 | -8 | 37 |
| Center 3 | 27 | 30 | 14 | 21 | 16 | 109 | 119 | 71 | 60 | 52 | 0 | 11 | -48 | -22 | -41 | 0 | 10 | -35 | -45 | -53 |
| Center 4 | 15 | 14 | 13 | 19 | 19 | 85 | 141 | 147 | 146 | 185 | 0 | -7 | -13 | 27 | 27 | 0 | 66 | 73 | 71 | 117 |
| Center 5 | 16 | 12 | 15 | 13 | 21 | 67 | 123 | 70 | 56 | 48 | 0 | -25 | -6 | -19 | 31 | 0 | 83 | 4 | -17 | -28 |
| Center 6 | 1 | 4 | 5 | 3 | 2 | 14 | 34 | 30 | 23 | 26 | 0 | 300 | 400 | 200 | 100 | 0 | 139 | 111 | 64 | 86 |
| Center 7 | 3 | 4 | 9 | 13 | 12 | 43 | 105 | 111 | 96 | 28 | 0 | 33 | 200 | 333 | 300 | 0 | 145 | 159 | 126 | -36 |
| Total / mean | 85 | 92 | 76 | 91 | 89 | 79 | 114 | 96 | 118 | 127 | 0 | 8 | -11 | 7 | 5 | 0 | 45 | 22 | 51 | 62 |
